# Atrial Fibrillation/Flutter in Transthyretin Cardiac Amyloidosis: Prevalence, Incidence, Clinical Predictors, and Effect of Tafamidis

**DOI:** 10.1016/j.jacadv.2025.102470

**Published:** 2025-12-24

**Authors:** Nicholas Chan, Yevgeniy Brailovsky, Sergio Teruya, Alfonsina Mirabal, Ariel Y. Weinsaft, Jeffeny De Los Santos, Samantha Guadalupe, Massiel Jimenez, Stephen Helmke, Margaret Cuomo, Dia Smiley, Angelo Biviano, Jose Dizon, Elaine Wan, Hirad Yarmohammadi, Mathew S. Maurer

**Affiliations:** a Seymour, Paul and Gloria Milstein Division of Cardiology, Department of Medicine, Columbia University Irving Medical Center and New York Presbyterian Hospital, New York, New York, USA; b Clinical Cardiovascular Research Laboratory for the Elderly, New York, New York, USA

**Keywords:** atrial fibrillation/flutter, ATTR-CA, tafamidis, transthyretin cardiac amyloidosis

## Abstract

**Background:**

Atrial fibrillation/flutter (AF) is common in transthyretin cardiac amyloidosis (ATTR-CA). The CHARGE-AF score has not been validated in ATTR-CA. The Columbia staging system is prognostic for survival, but its utility in predicting incident AF is unknown.

**Objectives:**

The authors aim to determine the predictors of prevalence and incidence of AF and the effect of tafamidis.

**Methods:**

This is a retrospective cohort study of 419 patients with ATTR-CA. AF was ascertained from review of electrocardiograms, extended rhythm, device interrogations, and charted history. Binary logistic regression assessed for factors associated with prevalent AF. Cox regression time-to-event analysis assessed for factors associated with incident AF.

**Results:**

AF was present in 58% (n = 244) of ATTR-CA at baseline. On multivariable logistic regression, higher Columbia score (OR: 1.48; 95% CI: 1.25-1.75) and higher left atrial volume index (LAVI) (OR: 1.05; 95% CI: 1.02-1.08) were associated with prevalent AF, whereas hereditary amyloid transthyretin (ATTRv) (OR: 0.19; 95% CI: 0.07-0.55) was protective (all *P* < 0.05). AF developed in 71 (41%) subjects without prior AF, over a median follow-up 2 years. On Cox regression, higher Columbia score (HR: 1.18; 95% CI: 1.01-1.38; *P* = 0.035) was associated with new onset AF, whereas CHARGE-AF and LAVI were not. ATTRv (HR: 0.44; 95% CI: 0.23-0.87; *P* = 0.017) and tafamidis (HR: 0.54; 95% CI: 0.30-0.95; *P* = 0.034) were protective.

**Conclusions:**

More than half of the ATTR-CA patients have AF, which was associated with wild-type amyloid transthyretin, greater Columbia stage, and increased LAVI. About half of the ATTR-CA individuals developed AF after 2 years, which was predicted by the Columbia score, but not the CHARGE-AF score. ATTRv and tafamidis were protective against incident AF.

Transthyretin cardiac amyloidosis (ATTR-CA) is an underappreciated etiology of diastolic heart failure in the older adult population. This form of restrictive cardiomyopathy is caused by degeneration of the normal tetrameric transthyretin protein complex from either aging (wild-type amyloid transthyretin [ATTRwt]) or inherited mutations (hereditary amyloid transthyretin [ATTRv]), which generates monomers that coalesce into insoluble, misfolded protein fibrils and subsequently deposit in tissues.^1^ Progressive infiltration of cardiomyocytes by amyloid fibrils in both atria and ventricles results in adverse chamber remodeling and fibrosis that potentiates development of tachyarrhythmias such as atrial fibrillation/flutter (AF), which have been observed in 48% to 88% in ATTR-CA cohorts.^2^, ^3^, ^4^, ^5^

Managing AF in ATTR-CA is challenging due to a lower rate of successful electrical cardioversion,^6^^,^^7^ and catheter ablation. Prior studies had conflicting results with regards to both safety/periprocedural complications and efficacy,^3^^,^^7^^,^^8^ with concerns of high chances for arrhythmia recurrence. However, greater success rates of rhythm control strategies seemed to be observed in earlier stage disease.^5^^,^^9^^,^^10^ Moreover, thromboembolic events represent major concerns as amyloid-mediated atrial mechanical dysfunction increases the risk of intracardiac thrombosis/systemic embolism^4^ and warrants timely initiation of prophylactic pharmacotherapy for stroke regardless of CHA_2_DS_2_Vasc Score.^11^

In light of these clinical considerations, there is growing interest in the role of clinical prediction models for early identification of ATTR-CA subjects with increased likelihood of having pre-existing or new onset AF. Prompt risk stratification can help determine which patients need more intensive surveillance for incident AF and timely initiation of appropriate anticoagulation. The CHARGE-AF score, previously derived from the general population,^12^^,^^13^ has not been validated in ATTR-CA. The Columbia staging system is prognostic for survival,^14^ but its utility in predicting incident AF is not known. Furthermore, the effect of disease-modifying therapy on the development of incident AF is unclear. The purpose of our study is multifold: 1) determine the prevalence, phenotypic characteristics, and relevant factors associated with pre-existing AF in ATTR-CA patients on presentation; 2) evaluate the incidence and clinical predictors for incident AF in ATTR-CA during follow-up, including the utility of CHARGE-AF score and Columbia staging system; 3) determine whether disease-modifying therapy with tafamidis delays the onset of incident AF.

## Methods

This is a single-center retrospective observational cohort study of 419 patients diagnosed with ATTR-CA from 2001 to 2021, with approval by the Institutional Review Board. Diagnosis of ATTR-CA was made by either endomyocardial biopsy or technetium pyrophosphate scan per the American Society of Nuclear Cardiology guidelines.^15^^,^^16^ Light chain amyloidosis was excluded by the absence of detectable monoclonal protein on serum and urine immunofixation electrophoresis, as well as normal serum-free light chain assay results, or biopsy.

The study cohort was first stratified into individuals with and without AF at baseline, as ascertained based on review of prior electrocardiograms (ECG), extended rhythm monitoring if performed, device interrogations when applicable, and documented history. For each of these 2 groups of subjects, baseline variables, including demographics, comorbidities, medications, implanted devices, vital signs, laboratory results, and echocardiographic measurements from standard clinical protocols, were collected on initial presentation. Hypertension, diabetes, coronary artery disease, myocardial infarction, stroke/transient ischemic attack, and smoking were based on charted history. Chronic kidney disease (CKD) was confirmed by history and an estimated glomerular filtration rate <60 mL/min/m^2^ per the modified diet in renal disease equation.

For cardiac biomarkers, when needed, high sensitivity troponin T and B-type natriuretic peptide were converted as follows: troponin T = hsTnT/1,000; NT-proBNP = BNP × 6, as we have performed previously.^14^ Staging of disease, which has prognostic implications on mortality, were determined as per previously established definitions (Supplemental Table 1).^14^^,^^17^^,^^18^ Left ventricular chamber dimensions and volumes (including stroke volume) as well as left ventricular mass and cardiac output were all indexed to body surface area. Myocardial contraction fraction was the ratio of left ventricular stroke volume to myocardial volume.^19^^,^^20^ Binary logistic (see statistical analysis section) was performed for the outcome of prevalent AF.

From the subcohort of individuals without any AF prior on initial presentation, we then identified which of these patients developed de novo AF on subsequent follow-up. The presence of incident AF was ascertained from review of charted history, ECGs, extended rhythm monitoring, and device interrogations. Clinical characteristics were compared between those with and without new onset AF, which was ascertained by the same methodology described previously. We also calculated each subject’s CHARGE-AF score (Supplemental Table 2) and recorded whether tafamidis was prescribed during any time point of these patients’ disease courses. Time-to-event analysis (see statistical analysis section) was performed for the endpoint of incident AF.

### Statistical analysis

Continuous variables were reported as medians with IQRs, whereas categorical variables were reported as number of patients followed by percentages. The differences in characteristics among groups were assessed by the Kruskal-Wallis test for continuous dependent variables and chi-Square test (or Fisher exact test) for nominal dependent variables.

Binary logistic regression was performed to determine factors associated with prevalent AF. Covariates were selected a priori to include age, race, ATTR genotype, cardiac amyloidosis staging, and left atrial (LA) volume index (LAVI), whereby all variables with *P* values <0.10 on univariable analysis were then entered into the multivariable model.

For incident AF, the date of initial presentation was defined as the beginning of the follow-up period. The end follow-up date was defined as the last date of clinical contact, death/heart transplant, or AF occurrence, whichever occurs first, as of September 30, 2022. Time-to-event analysis, with censoring after 5-years of follow-up, was performed using Cox proportional hazard modeling to assess the effects of relevant factors on the development of new onset AF. Covariates were selected a priori based on their clinical relevance, to include CHARGE-AF score (which already incorporates age and race), gender, ATTR genotype, cardiac amyloidosis staging, LAVI, and tafamidis use. All variables with *P* values <0.10 in the univariable analysis were included in the multivariable model. All statistical tests were 2-sided, with a significance level set at α = 0.05. All statistical analyses were completed in IBM SPSS Statistics (version 28) (2021).

To test the proportional hazards (PHs) assumption, time-dependent covariates (as interaction terms between each covariate and the log of time) were added to the original Cox PH model and found nonsignificant *P* values (*P* > 0.05) for all the time interaction terms. In addition, a supremum test and cumulative Martingale residuals (based on Schoenfeld residuals) further tested the PH assumption of the continuous covariates. The supremum test showed *P* values of 0.13 and 0.55 for the Columbia score and LAVI respectively, and the cumulative Martingale residuals plots did not show any trend over time, demonstrating that the PH assumption was held. Collinearity was not found; the highest variance inflation factor found was for the ATTRv variable, 1.3 (>10 suggests collinearity) and condition indices (CIs) were all below 16 (>30 suggests collinearity).

## Results

Among the 419 study subjects, 175 (42%) had no prior AF, whereas 244 (58%) had pre-existing AF (prevalent AF cohort) (Table 1). Compared to the no AF group, the prevalent AF cohort was older (78 years vs 74 years), had more individuals of White race (84.0% vs 68.6%), but fewer of Hispanic ethnicity (2.5% vs 7.4%) and fewer with ATTRv (13.1% vs 39.4%). Those with prevalent AF had more advanced NYHA functional class (III to IV symptoms of 48.7% vs 33.8%), prior stroke/transient ischemic attack (14.8% vs 5.7%), CKD (65.6% vs 44.6%), and need for diuretic agents (82.0% vs 65.7%). The median follow-up for the no baseline AF cohort is 3.58 (95% CI: 2.86-4.20) years. As expected, those with prevalent AF were more frequently treated with various antiarrhythmic medications and anticoagulation (and less frequently on aspirin). Labs show that the prevalent AF group had worse renal function and N-terminal pro–B-type natriuretic peptide levels than the no AF cohort. The cohort with AF at baseline also had greater proportion of individuals having intermediate to advanced (stage II to III) disease, per National Amyloidosis Centre (NAC) (47.1% vs 31.6%) and Columbia (70.3% vs 48.9%) staging systems. Echocardiographic features were similar between the 2 groups, except that the prevalent AF group had greater LA diameter (4.7 cm vs 4.4 cm) and LAVI (48.1 mL/m^2^ vs 40.9 mL/m^2^).

**Table 1:** Baseline Characteristics of Study Cohort (N = 419)

	n	No AF (n = 175, 42%)	n	Prevalent AF (n = 244, 58%)	*P* Value
Demographics					
Age, y	175	74 (68, 79)	244	78 (73, 82)	<0.0001
Male	175	148 (84.6%)	244	215 (88.1%)	0.293
White race	175	120 (68.6%)	244	205 (84.0%)	<0.0001
ATTRv	175	69 (39.4%)	244	32 (13.1%)	<0.0001
Hispanic ethnicity	175	13 (7.4%)	244	6 (2.5%)	0.016
Body mass index, kg/m^2^	174	26.2 (23.8, 28.7)	244	26.5 (24.1, 29.7)	0.283
NYHA functional class	175		244		<0.0001
I		28 (16.0%)		14 (5.7%)	
II		88 (50.3%)		111 (45.5%)	
III		54 (30.9%)		114 (46.7%)	
IV		5 (2.9%)		5 (2.0%)	
Comorbidities					
Hypertension	175	91 (52.0%)	244	125 (51.2%)	0.876
Diabetes	175	18 (10.3%)	244	30 (12.3%)	0.524
CAD	175	52 (29.7%)	244	92 (37.7%)	0.089
MI	175	11 (6.3%)	244	18 (7.4%)	0.664
Stroke/TIA	175	10 (5.7%)	244	36 (14.8%)	0.004
CKD	175	78 (44.6%)	244	160 (65.6%)	<0.0001
Smoking	175		244		0.122
Active		6 (3.4%)		2 (0.8%)	
Former		66 (37.7%)		103 (42.2%)	
Never		103 (58.9%)		139 (57.0%)	
Antiarrhythmics					
Beta-blocker	175	70 (40.0%)	244	151 (61.9%)	<0.0001
Amiodarone	175	3 (1.7%)	244	33 (13.5%)	<0.0001
Dofetilide	175	0 (0%)	244	6 (2.5%)	0.043
Calcium channel blockers	175	13 (7.4%)	244	21 (8.6%)	0.663
Digoxin	175	2 (1.1%)	244	14 (5.7%)	0.016
Heart failure therapy					
ACEI/ARB/ARNI	175	64 (36.6%)	244	79 (32.4%)	0.372
MRA	175	45 (25.7%)	244	80 (32.8%)	0.119
Loop diuretic agents	175	115 (65.7%)	244	200 (82.0%)	<0.0001
Thiazides	175	20 (11.4%)	244	29 (11.9%)	0.886
Antiplatelet					
ASA	175	91 (52.0%)	244	69 (28.3%)	<0.0001
Clopidogrel	175	14 (8.0%)	244	13 (5.3%)	0.272
Ticagrelor	175	0 (0%)	244	1 (0.4%)	1.000
Prasugrel	175	2 (1.1%)	244	0 (0%)	0.174
Ticlopidine	175	0 (0%)	244	1 (0.4%)	1.000
Dipyridamole	175	0 (0%)	244	1 (0.4%)	1.000
Anticoagulation					
Warfarin	175	5 (2.9%)	244	72 (29.5%)	<0.0001
DOAC	175	3 (1.7%)	244	140 (57.4%)	<0.0001
Other CV medications					
Statin	175	99 (56.6%)	244	148 (60.7%)	0.402
Devices					
PPM only	175	9 (5.1%)	244	39 (16.0%)	<0.0001
ICD	175	17 (9.7%)	244	34 (13.9%)	0.193
Vitals					
Systolic blood pressure, mm Hg	173	120 (108, 130)	244	117 (104, 126)	0.187
Diastolic blood pressure, mm Hg	173	70 (64, 80)	244	70 (60, 80)	0.207
Heart rate, beats/min	173	73 (64, 82)	244	76 (65, 82)	0.724
Labs					
Creatinine (mg/dL)	175	1.17 (0.95, 1.45)	244	1.32 (1.08, 1.70)	<0.0001
eGFR (mL/min/m^2^)	174	63 (49, 77)	244	53 (40, 67)	<0.0001
troponin T (ng/L)	157	0.05 (0.03, 0.11)	220	0.05 (0.02, 0.10)	0.682
NT-proBNP (pg/mL)	174	1885 (914, 4,039)	240	2,623 (1,748, 4,641)	<0.0001
Amyloidosis staging					
Mayo	168		233		0.868
1		96 (57.1%)		139 (59.7%)	
2		40 (23.8%)		51 (21.9%)	
3		32 (19.0%)		43 (18.5%)	
Gilmore	174		240		0.005
1		119 (68.4%)		127 (52.9%)	
2		32 (18.4%)		59 (24.6%)	
3		23 (13.2%)		54 (22.5%)	
Columbia	174		239		<0.0001
Stage I (1-3 points)		89 (51.1%)		71 (29.7%)	
Stage II (4-6 points)		69 (39.7%)		127 (53.1%)	
Stage III (7-9 points)		16 (9.2%)		41 (17.2%)	
Echo					
LVEF, %	140	49 (33, 55)	190	50 (35, 55)	0.959
IVS, cm	138	1.7 (1.5, 1.9)	181	1.6 (1.4, 1.8)	0.056
PWT, cm	135	1.6 (1.4, 1.8)	177	1.5 (1.3, 1.8)	0.399
RWT	134	0.73 (0.59, 0.88)	175	0.70 (0.52, 0.88)	0.401
LVEDDi, cm/m^2^	136	2.3 (2.1, 2.5)	175	2.3 (2.0, 2.5)	0.446
LVESDi, cm/m^2^	134	1.8 (1.6, 2.2)	175	1.8 (1.5, 2.1)	0.504
LVEDVi, mL/m^2^	136	44 (38, 54)	175	44 (36, 53)	0.784
LVESVi, mL/m^2^	134	23 (17, 32)	175	23 (17, 31)	0.769
LVSVi, mL/m^2^	134	20 (14, 25)	175	21 (16, 25)	0.596
LV mass index, g/m^2^	132	154 (127, 191)	172	148 (120, 180)	0.099
Cardiac index, L/min/m^2^)	133	1.5 (1.1, 1.8)	175	1.5 (1.2, 1.9)	0.473
MCF, %	132	13 (9, 18)	173	14 (10, 20)	0.167
LA diameter (cm)	148	4.4 (4.0, 4.8)	201	4.7 (4.2, 5.0)	<0.0001
LAVI (mL/m^2^)	132	40.9 (34.9, 48.2)	168	48.1 (40.5, 56.6)	<0.0001

ACEI = angiotensin-converting enzyme inhibitor; AF = atrial fibrillation/flutter; ARB = angiotensin receptor blocker; ARNI = angiotensin receptor neprilysin inhibitor; ASA = aspirin; ATTRv = hereditary amyloid transthyretin; CAD = coronary artery disease; CKD = chronic kidney disease; CV = cardiovascular; DOAC = direct oral anticoagulant; eGFR = estimated glomerular filtration rate; ICD = implantable cardioverter-defibrillator; IVS = intraventricular septal thickness; LVEDDi = left ventricular diastolic dimension index; LVEDVi = left ventricular diastolic volume index; LVESDi = left ventricular systolic dimension index; LVESVI = left ventricular stroke volume index; MCF = myocardial contraction fraction; MI = myocardial infarction; MRA = mineralocorticoid receptor antagonist; NT-proBNP = N-terminal pro–B-type natriuretic peptide; LA = left atrial; LAVI = left atrial volume index; LV = left ventricular; LVEF = left ventricular ejection fraction; PPM = permanent pacemaker; PWT = posterior wall thickness; RWT = relative all thickness; TIA = transient ischemic attack.

Binary logistic regression model for the effect of clinically relevant covariates on the presence of pre-existing AF is shown in Table 2. On multivariable analysis, Columbia score (OR: 1.48; 95% CI: 1.25-1.75; *P* < 0.0001, per point increase in score) and LAVI (OR: 1.05; 95% CI: 1.02-1.08; *P* < 0.0001, per mL/m^2^ increase) were associated with greater odds of prevalent AF, whereas ATTRv had lower odds of prevalent AF (OR: 0.19; 95% CI: 0.07-0.55; *P* = 0.002) when compared to ATTRwt.

**Table 2:** Baseline Characteristics of Subjects Without Any Prior AF On Initial Presentation, Stratified by Those Who Develop and Do Not Develop Incident AF During Follow-Up (N = 175)

	n	No Incident AF (n = 104, 59%)	n	Incident AF (n = 71, 41%)	*P* Value
Demographics					
Age, years	104	74 (66, 80)	71	75 (71, 79)	0.505
Male	104	82 (78.8%)	71	66 (93.0%)	0.011
White race	104	64 (61.5%)	71	56 (78.9%)	0.015
ATTRv	104	51 (49.0%)	71	18 (25.4%)	0.002
Hispanic ethnicity	104	11 (10.6%)	71	2 (2.8%)	0.055
Height, cm	103	173 (165, 178)	71	173 (165, 180)	0.217
Weight, kg	103	76 (68, 85)	71	82 (70, 90)	0.057
Body mass index, kg/m^2^	103	26.0 (23.4, 28.2)	71	26.4 (24.0, 29.7)	0.233
NYHA functional class	104		71		0.022
I		22 (21.2%)		6 (8.5%)	
II		47 (45.2%)		41 (57.7%)	
II		30 (28.8%)		24 (33.8%)	
IV		5 (4.8%)		0 (0.0%)	
CHARGE-AF score	104	14.0 (13.2, 14.7)	71	14.3 (13.6, 14.8)	0.064
Comorbidities					
Hypertension	104	61 (58.7%)	71	30 (42.3%)	0.033
Diabetes	104	12 (11.5%)	71	6 (8.5%)	0.509
CAD	104	33 (31.7%)	71	19 (26.8%)	0.48
MI	104	7 (6.7%)	71	4 (5.6%)	1.000
Stroke/TIA	104	3 (2.9%)	71	7 (9.9%)	0.093
CKD	104	44 (42.3%)	71	34 (47.9%)	0.466
Smoking	104		71		0.42
Active		2 (1.9%)		4 (5.6%)	
Former		39 (37.5%)		27 (38.0%)	
Never		63 (60.6%)		40 (56.3%)	
Antiarrhythmics					
Beta-blocker	104	40 (38.5%)	71	30 (42.3%)	0.615
Amiodarone	104	3 (2.9%)	71	0 (0%)	0.273
Calcium channel blockers	104	7 (6.7%)	71	6 (8.5%)	0.67
Digoxin	104	1 (1.0%)	71	1 (1.4%)	1.000
Heart failure therapy					
ACEI/ARB/ARNI	104	38 (36.5%)	71	26 (36.6%)	0.991
MRA	104	24 (23.1%)	71	21 (29.6%)	0.334
Loop diuretic agents	104	65 (62.5%)	71	50 (70.4%)	0.278
Thiazides	104	10 (9.6%)	71	10 (14.1%)	0.362
Antiplatelet					
ASA	104	54 (51.9%)	71	37 (52.1%)	0.98
Clopidogrel	104	7 (6.7%)	71	7 (9.9%)	0.454
Prasugrel	104	0 (0%)	71	2 (2.8%)	0.163
Anticoagulation					
Warfarin	104	3 (2.9%)	71	2 (2.8%)	1.000
DOAC	104	1 (1.0%)	71	2 (2.8%)	0.567
Other CV medications					
Statin	104	56 (53.8%)	71	43 (60.6%)	0.379
Devices					
PPM only	104	3 (2.9%)	71	6 (8.5%)	0.161
ICD	104	11 (10.6%)	71	6 (8.5%)	0.641
Vitals					
Systolic blood pressure, mm Hg	102	120 (106, 130)	71	119 (110, 130)	0.698
Diastolic blood pressure, mm Hg	102	70 (62, 78)	71	70 (65, 80)	0.103
Heart rate, bpm	104	73 (64, 83)	71	73 (65, 80)	0.686
Labs					
Creatinine (mg/dL)	104	1.16 (0.94, 1.45)	71	1.17 (1.00, 1.45)	0.511
eGFR (mL/min/m^2^)	103	64 (50, 77)	71	62 (46, 76)	0.521
troponin T (ng/L)	99	0.05 (0.02, 0.09)	70	0.05 (0.03, 0.12)	0.319
NT-proBNP (pg/mL)	103	1830 (780, 3,837)	71	1904 (1,081, 4,257)	0.173
Amyloidosis staging					
Mayo	98		70		0.987
1		56 (57.1%)		40 (57.1%)	
2		23 (23.5%)		17 (24.3%)	
3		19 (19.4%)		13 (18.6%)	
Gilmore	103		71		0.599
1		70 (68.1%)		49 (69.0%)	
2		21 (20.4%)		11 (15.5%)	
3		12 (11.7%)		11 (15.5%)	
Columbia	103		71		0.557
Stage I (1-3 points)		54 (52.4%)		35 (49.3%)	
Stage II (4-6 points)		38 (36.9%)		31 (43.7%)	
Stage III (7-9 points)		11 (10.7%)		5 (7.0%)	
Echo					
LVEF, %	82	47 (34, 56)	58	50 (33, 55)	0.786
IVS, cm	81	1.6 (1.4, 2.0)	57	1.7 (1.5, 1.9)	0.725
PWT, cm	79	1.5 (1.3, 1.8)	56	1.6 (1.4, 1.8)	0.469
RWT	78	0.71 (0.56, 0.90)	56	0.74 (0.60, 0.86)	0.649
LVEDDi, cm/m^2^	80	2.3 (2.0, 2.6)	56	2.2 (2.1, 2.4)	0.324
LVESDi, cm/m^2^	78	1.8 (1.6, 2.2)	56	1.8 (1.5, 2.1)	0.711
LVEDVi, mL/m^2^	80	46 (37, 56)	56	43 (39, 50)	0.573
LVESVi, mL/m^2^	78	24 (17, 33)	56	22 (18, 31)	0.896
LVSVi, mL/m^2^	78	19 (14, 28)	56	21 (14, 25)	0.921
LV mass index, g/m^2^	77	162 (126, 193)	55	151 (131, 181)	0.664
Cardiac index, L/(min∗m^2^)	77	1.4 (1.1, 1.8)	56	1.5 (1.1, 1.8)	0.989
MCF, %	77	12 (9, 19)	55	14 (9, 17)	0.93
LAVI (mL/m^2^)	78	39.5 (34.0, 48.3)	64	41.5 (36.6, 48.2)	0.167
Disease modifying agents on follow-up					
Tafamidis	104	56 (53.8%)	71	39 (54.9%)	0.888
Diflunisal	104	16 (15.4%)	71	17 (23.9%)	0.155

Abbreviation as in Table 1.

**Table 3:** Binary Logistic Regression for Pre-Existing AF on Initial Presentation

		95.0% CI		
Univariable Model	OR	Lower	Upper	*P* Value
Age, per year	1.054	1.028	1.080	<0.001
White race (reference: non-White)	2.409	1.509	3.847	<0.001
ATTRv (reference: ATTRwt)	0.232	0.144	0.375	<0.001
Hispanic (reference: non-Hispanic)	0.314	0.117	0.844	0.022
Columbia score, per point	1.305	1.169	1.457	<0.001
LAVI, per mL/m^2^	1.064	1.039	1.089	<0.001

Multivariable Model				
Age, per year	1.029	0.992	1.067	0.126
White race (reference: non-White)	0.791	0.297	2.107	0.639
ATTRv (reference: ATTRwt)	0.193	0.068	0.547	0.002
Hispanic (reference: non-Hispanic)	0.577	0.157	2.119	0.407
Columbia score, per point	1.482	1.253	1.752	<0.001
LAVI, per mL/m^2^	1.048	1.022	1.075	<0.001

From the total of 419 (total study sample size), 298 observations were used in the multivariate logistic regression model. 121 observations were removed due to missing values for explanatory variables.

ATTRwt = wild-type amyloid transthyretin; other abbreviations as in Table 1.

Of the 175 subjects who had no prior AF at baseline, 104 (59%) did not developed de novo AF (no incident AF cohort) and 71 (41%) developed new onset AF (incident AF cohort) during the follow-up (Table 3). The presence of AF was determined from serial ECGs in 56 (78.9%) patients, cardiac device interrogation in 10 (14.1%) patients, and extended rhythm monitoring in 5 (7.0%) patients. Compared to the no incident AF group, the incident AF cohort had more individuals of male sex (93.0% vs 78.8%) and White race (78.9% vs 61.5%) but fewer with a genetic variant of ATTR (25.4% vs 49.0%), NYHA class I functional status (8.5% vs 21.2%), and history of hypertension (42.3% vs 58.7%). From the 175 subjects with no prevalent AF, 18 (10.3%) underwent heart transplant and 67 (38.3%) died. There were no major differences in age, ethnicity, body habitus, CHARGE-AF score, other comorbidities, implanted devices, vital signs, labs, or echocardiographic findings between the 2 groups.

Cox proportional modeling for the effect of clinically relevant covariates on incident AF is shown in Table 4. The median follow-up time was 2.0 years for the overall subcohort, 1.9 years for the incident AF cohort, and 2.5 years for the no incident AF cohort. On multivariable analysis, per point increase in Columbia score (HR: 1.18; 95% CI: 1.01-1.38; *P* = 0.035) was predictive of incident AF, whereas CHARGE-AF was not significant even on the univariable model. Moreover, ATTRv (HR: 0.44; 95% CI: 0.23-0.87; *P* = 0.017) and tafamidis (HR: 0.54; 95% CI: 0.30-0.95; *P* = 0.034) were protective.

**Table 4:** Cox Proportional Hazard Modeling for Endpoint of Incident AF

		95.0% CI		
Univariable Cox Regression	HR	Lower	Upper	*P* Value
Male (reference: female)	2.545	1.025	6.320	0.044
ATTRv (reference: ATTRwt)	0.532	0.311	0.910	0.021
Hispanic (reference: non-Hispanic)	0.398	0.097	1.626	0.199
CHARGE-AF score, per point	1.200	0.950	1.515	0.125
Columbia score, per point	1.182	1.043	1.340	0.009
LAVI, per mL/m^2^	1.038	1.011	1.065	0.005
Tafamidis	0.664	0.415	1.062	0.087

Multivariable Cox Regression				
Male (reference: female)	1.75	0.600	5.119	0.305
ATTRv (reference: ATTRwt)	0.44	0.225	0.865	0.017
Columbia score, per point	1.18	1.012	1.384	0.035
LAVI, per mL/m^2^	1.03	0.999	1.051	0.058
Tafamidis	0.54	0.301	0.954	0.034

From the total of 175 with no prior AF on initial presentation, 132 observations were used in the multivariate Cox PH model. 43 observations were removed due to missing values for explanatory variables.

Abbreviation as in Tables 1 and 2.

## Discussion

This study explored the clinical factors associated with prevalent AF at initial presentation and predictors of de novo AF during the follow-up for patients diagnosed with ATTR-CA. The major findings are: 1) ATTRwt, greater Columbia score, and dilated LA are most closely associated with pre-existing AF; and 2) the Columbia score, but not the CHARGE-AF score, was predictive of new onset AF, whereas ATTRv and tafamidis were protective against it (Central Illustration).

**Central Illustration:**
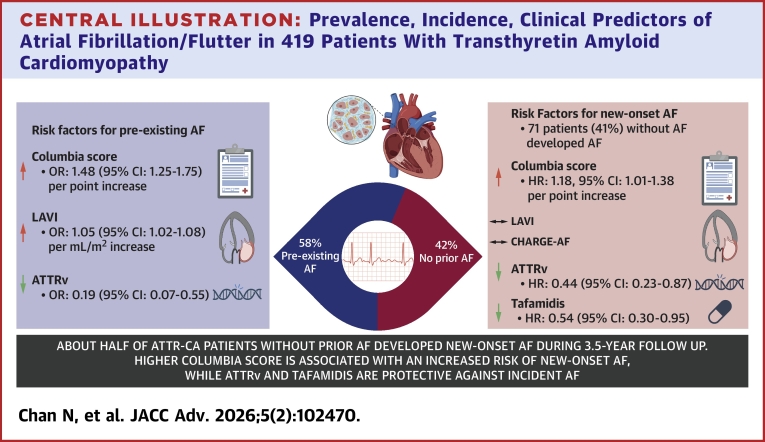
**Prevalence, Incidence, Clinical Predictors of Atrial Fibrillation/Flutter in 419 Patients With Transthyretin Amyloid Cardiomyopathy** Created in BioRender, Brailovsky Y (2025), https://BioRender.com/btmxrvj. AF = atrial fibrillation/flutter; ATTR = transthyretin amyloid; LAVI = left atrial volume index; ATTRv = hereditary amyloid transthyretin; ATTR-CA = transthyretin cardiac amyloidosis.

Prior literature has reported an AF prevalence of about 50 to 88% in ATTR-CA(2-5), of which our study’s prevalence falls within the previously reported range (at initial presentation: 58% [244/419]; overall final rate when adding the 71 incident AF cases: 75% [315/419]). Most recently, Papathanasiou et al^2^ performed a retrospective cohort study of 133 cardiac amyloidosis patients seen at their tertiary care center over a 15-year period. Their overall AF prevalence was 71%, with 80% (43/54) of ATTRwt and 12.5% (1/8) of ATTRv having pre-existing AF. Although these were slightly different from our study population (67% [212/318] of ATTRwt and 32% [32/101] of ATTRv), we both show that proportionally more ATTRwt than ATTRv had AF at baseline.^2^ This difference of AF prevalence between ATTRwt and ATTRv may be more reflective of the difference in prevalence of AF in varying races rather than reflective of underlying pathophysiology of ATTR-CA. Patients with ATTRwt are predominantly White, whereas ATTRv are predominantly black in our cohort. It has been previously demonstrated that in general population AF prevalence is substantially higher in White compared to Black patients.^21^

Similar to our cohort, prior studies showed that subjects with prevalent AF, when compared to those with no AF, were older individuals who more often had NYHA functional class III to IV functional status, CKD, and greater LAVI. We also demonstrate increasing prevalence of AF with worsening ATTR-CA disease severity per NAC staging and Columbia staging, the latter of which incorporates NAC stage + NYHA functional class + diuretic dose (NAC stage I: 52% [127/246], NAC stage II: 65% [59/91], NAC stage III: 70% [54/77]; Columbia stage I: 44% [71/160]; Columbia stage II: 65% [127/196]; Columbia stage III: 72% [41/57]).

In contrast to the known high prevalence of AF, the real incidence during the follow-up remains unclear. Ascertainment of de novo AF is challenging. AF can be documented on routine ECG during follow-up or only assessed for symptomatic episodes. Additionally prolonged monitoring can be used to assess for paroxysmal AF. The mode and duration of monitoring would yield varying incidence of AF, with longer duration of monitoring with 30-day event monitor and implantable loop recorder likely associated with higher yield, as has been previously shown in patients with stroke.^22^^,^^23^ Using a combination of ECGs, device interrogations and long-term rhythm monitoring could introduce an ascertainment bias as not all subjects underwent long-term monitoring and only those with devices have them interrogated. Those subjects with more assessments for incident AF (e.g. electrocardiogram, long term monitoring and/or device interrogation) are more likely to have it detected. Such strategy is a strength of our study in that we evaluated for incident AF using a multimodal approach, which differs from other studies in which incident AF is only detected by ECGs.

Ascertainment of de novo AF was first addressed by Donnellan et al.^5^ in a retrospective cohort study of 382 ATTR-CA patients (71% ATTRwt, 29% ATTRv). They found that 60.2% patients developed incident AF and older age (HR: 1.02 per year; 95% CI: 1.005-1.03; *P* = 0.007), advanced NAC ATTR-CA stage (HR: 1.18; 95% CI: 1.005-1.39; *P* = 0.04), and higher LAVI (HR: 1.01 per ml/m^2^; 95% CI: 1-1.02; *P* = 0.045) to be associated with developing AF,^5^ similar to our findings. In our study, we ascertained the presence of AF via several methods, including cardiac device interrogation in 35% of patients, extended outpatient rhythm monitoring in 54% of patients, and serial ECGs in 11% of patients.

A more recent investigation was performed by a multicenter retrospective observational cohort study by Fumagalli et al.^24^ which consisted of 266 consecutive ATTRwt patients. In this population, history of AF was confirmed by the review of prior clinical documentation of arrhythmia episodes, and all patients had at least annual Holter monitoring. In their study, 29% subjects developed de novo AF,^24^ with a median time to AF onset of 27 months.^24^ On multivariable Fine-Gray Regression analysis, with mortality as a competing risk for incidence of de novo AF, they demonstrate several markers on baseline ECG to be predictive of incident AF (increased PR interval and QRS duration) as well as increased LA diameter.^24^ Patients with at least 2 risk factors (PR ≥ 200 ms, QRS ≥120 ms, or LA diameter ≥50 mm) had the highest risk of developing de novo AF compared to patients without risk factors (HR: 14.918; 95% CI: 3.242-31.646; *P* = 0.008). Although this represented the first study to directly associate ECG parameters to de novo AF in ATTRwt, staging was not able to be performed due to missing biomarkers.

The incidence of AF in our study was 41% (71/175 subjects without prior AF), which falls in the middle of the values reported by these 2 previous studies.^5^^,^^24^ We confirmed the predictive utility of ATTR-CA staging for incident AF via the Columbia score, of which per point increase led to a 18% greater risk for incident AF, but LAVI or LA diameter failed to reach significance. What is intriguing and not looked at by either of the prior investigations, is the lack of utility for the CHARGE-AF score for ATTR-CA patients despite its value in predicting de novo AF in the general population. This may be explained by the fact that all the ATTR-CA patients all had heart failure and are likely sicker than general population with AF thereby lowering its discriminatory ability. ATTRv was also shown to be strongly protective against incident AF, although unclear if this could partially be attributed to lower age. In addition, a cross-sectional analysis of the MESA (Multiethnic Study of Atherosclerosis) study has demonstrated that the prevalence of clinically detected AF is significantly lower in African American compared to in White patients,^25^ which may explain the protective effect of ATTRv in our cohort, most of whom are African Americans with Val142Ile mutation. However, in that same study, unbiased AF detection using ambulatory monitoring in the same individuals revealed minimal difference in the proportion with AF by race.^25^ In our study, a multimodal approach for AF detection was used to minimize such bias.

Moreover, given that disease-modifying therapy was known to be prescribed to most of our patients (54.3% [95/175]) unlike prior studies, we were able to perform an analysis of the effect of tafamidis on incident AF. We found that tafamidis exerted a strong protective effect with an estimated 46% reduction in the incident AF. This is consistent with findings from a prior retrospective study of ATTR-CA patients from our center, which showed that after adjustment for measured confounders including age, sex, left ventricular ejection fraction, obesity, hypertension, and LA diameter, tafamidis use reduced the risk of incident AF development by 57%, *P* = 0.03.^26^ Prior imaging studies have demonstrated tafamidis delays structural and functional changes in the heart, as reflected by lesser deterioration of biventricular ejection fraction and global longitudinal strain, LA reservoir strain, left ventricular mass index, and extracellular volume.^27^, ^28^, ^29^ These effects may in part mediate the observation that tafamidis use is associated with lower incidence of atrial fibrillation. Notably, in the ATTR-ACT (Tafamidis in Transthyretin Cardiomyopathy Clinical Trial) trial, although events were very low, tafamidis was associated with a lower risk of hospitalization for stroke/transient ischemic attack (2.7% vs 4.5%) and lower risk of hospitalization for arrhythmias (15.2% vs 21.5%).^30^

### Study Limitations

There are several limitations to our investigation. First, this is a single-center retrospective observational cohort study, with inherent selection bias and residual confounding in our modeling that prevents definitive conclusions about which clinical factors are the most important in determining AF development. Second, AF was assessed as either present or absent, without accounting for duration of episodes, which may be important in some cases with regards to clinical significance. Third, despite our thorough approach to assess for AF, occult low-burden AF may have been missed since not all subjects had long-term monitoring or implanted devices and patients with paroxysmal and asymptomatic AF are most likely to be missed. Fourth, we did not collect baseline electrocardiographic parameters on all individuals and did not explore ECG-derived predictors of future AF, but this is of interest and should be explored in future studies. Fifth, LA strain was not routinely assessed. We did not assess the impact of sodium glucose co-transporter 2 inhibitors on patient outcomes, as a substantial proportion of patients were enrolled before approval and wide implementation of this class of medications. Tafamidis use was determined at baseline, and it was not included in the model as a time-varying covariate. Finally, the limited duration and modality of AF monitoring may have led to underestimation of incident AF cases, particularly if AF was paroxysmal or asymptomatic. Further research with multicenter subject pools would be helpful to validate our results.

## Conclusions

More than half of ATTR-CA patients have pre-existing AF on initial presentation, which is most closely associated with ATTRwt, greater Columbia stage, and dilated LA. About half of the ATTR-CA patients without prior AF subsequently developed new onset AF during 3.5 year follow-up, which was predicted by the Columbia score but not CHARGE-AF score. ATTRv and tafamidis were protective against incident AF.

## Funding support and author disclosures

Dr Maurer has received grant support from 10.13039/100000002NIH R01 HL139671 and R01AG081582-01; consulting income from BridgeBio, Ionis, Novo-Nordisk, Pfizer, Astra Zeneca, Alnylam, and Intellia; and institutional support in the form of clinical trial funding from Pfizer, Attralus, Ionis, BridgeBio, Intellia, and Alnylam. Dr Brailovsky has received consulting fees from BridgeBio, AstraZeneca, Pfizer, and Alnylam. Dr Yarmohammadi has received consulting fee from Biotronik. All other authors have reported that they have no relationships relevant to the contents of this paper to disclose.
