# Supplementary material for: Atrial Fibrillation/Flutter in Transthyretin Cardiac Amyloidosis: Prevalence, Incidence, Clinical Predictors, and Effect of Tafamidis
Source: JACC Adv. 2025 Dec 24;5(2):102470. doi: 10.1016/j.jacadv.2025.102470 (PMC12800393; doi:10.1016/j.jacadv.2025.102470)
Supplement: Supplemental data [file mmc1.docx]

**Supplementary Material**

**Table S1. Definitions of Mayo, National Amyloidosis Center (NAC), Columbia Staging Systems.**

| **Mayo** |  |
| --- | --- |
| *Stage I* | Troponin T < 0.05 and NT-proBNP < 3000 pg/mL (0 points) |
| *Stage II* | Troponin T ≥ 0.05 OR NT-proBNP ≥ 3000 pg/mL (1 point) |
| *Stage III* | Troponin T ≥ 0.05 AND NT-proBNP ≥ 3000 pg/mL (2 points) |
|  | |
| **NAC** |  |
| *Stage I* | eGFR ≥ 45 mL/min and NT-proBNP < 3000 pg/mL (0 points) |
| *Stage II* | eGFR < 45 mL/min OR NT-proBNP ≥ 3000 pg/mL (1 point) |
| *Stage III* | eGFR < 45 mL/min AND NT-proBNP ≥ 3000 pg/mL (2 points) |
|  | |
| **Columbia Score (range 1-9)** | = NAC or Mayo stage as above with added points + diuretic dose + NYHA class |
| *Columbia Stage I*  *Columbia Stage II*  *Columbia Stage III* | Columbia score: 1-3 points  Columbia score: 4-6 points  Columbia score: 7-9 points |
| Diuretic Dose | |
| 0 points  1 point  2 points  3 points | 0 mg/kg of furosemide daily  >0 to 0.5 mg/kg of furosemide daily  >0.5 to 1 mg/kg of furosemide daily  >1 mg/kg of furosemide daily |
| NYHA Class | |
| 1 point  2 points  3 points  4 points | NYHA functional class I  NYHA functional class II  NYHA functional class III  NYHA functional class IV |

**Table S2. Calculation of the CHARGE-AF score.**

| Age | 0.508 x (per 5 yrs) |
| --- | --- |
| White Race | 0.465 |
| Height | 0.248 x (per 10 cm) |
| Weight | 0.115 x (per 15 kg) |
| Systolic Blood Pressure | 0.197 x (per 20 mm Hg) |
| Diastolic Blood Pressure | -0.101 x (per 10 mm Hg) |
| Current Smoker | 0.359 |
| Anti-Hypertensive Medications | 0.349 |
| Type II Diabetes | 0.237 |
| Congestive Heart Failure | 0.701 |
| Myocardial Infarction | 0.496 |
